# Progress in the cryoablation and cryoimmunotherapy for tumor

**DOI:** 10.3389/fimmu.2023.1094009

**Published:** 2023-01-25

**Authors:** Zenan Chen, Liangliang Meng, Jing Zhang, Xiao Zhang

**Affiliations:** ^1^ Department of Radiology, The First Medical Center, Chinese People's Liberation Army (PLA) General Hospital, Beijing, China; ^2^ Department of Radiology, Chinese People's Armed Police (PAP) Force Hospital of Beijing, Beijing, China

**Keywords:** cryoablation, immunotherapy, combination therapy, tumors, progress, cryoimmunotherapy

## Abstract

With the rapid advancement of imaging equipment and minimally invasive technology, cryoablation technology is being used more frequently in minimally invasive treatment of tumors, primarily for patients with early tumors who voluntarily consent to ablation as well as those with advanced tumors that cannot be surgically removed or cannot be tolerated. Cryoablation is more effective and secure for target lesions than other thermal ablation methods like microwave and radiofrequency ablation (RFA). The study also discovered that cryoablation, in addition to causing tumor tissue necrosis and apoptosis, can facilitate the release of tumor-derived autoantigens into the bloodstream and activate the host immune system to elicit beneficial anti-tumor immunological responses against primary. This may result in regression of the primary tumor and distant metastasis. The additional effect called “ Accompanying effects “. It is the basis of combined ablation and immunotherapy for tumor. At present, there is a lot of research on the mechanism of immune response induced by cryoablation. Trying to solve the question: how positively induce immune response. In this review, we focus on: 1. the immune effects induced by cryoablation. 2. the effect and mechanism of tumor immunotherapy combined with cryoablation. 3.The clinical research of this combination therapy in the treatment of tumors.

## Introduction

What is Cryoablation? Although the use of cryotherapy for fractures and wounds dates back to 3000 BC, the first application of cryotherapy for tumors was performed by Dr. James Arnott in the mid-19th century (1), he used crushed ice salt solutions of -18°C to -24°C for cryotherapy of breast, cervical, and skin cancers. He observed varying degrees of shrinkage of these frozen tumors and varying degrees of pain reduction in patients. Thus, cryoablation began to develop rapidly over time.

At present, the cryoablation technology in clinical applications includes mainly liquid or gas as the medium. Among them, the cryoablation equipment with gas as the medium has high efficiency, and the representative one is the argon-helium knife system. It’s working principle is based on gas throttling effect (Joule - Thomson effect), in which high-pressure argon gas flows through a small hole and quickly enters a low-pressure space, rapidly expands and absorbs the surrounding heat. The local temperature of the cryoablation needle quickly dropped below -140°C, which caused the formation of ice crystals inside and outside the tumor cells. The rupture of the cell membrane, and the occlusion of micro vessels in the ablation zone, leads to tumor cell ischemia and necrosis. At the same time, the temperature of the probe could be raised from -140°C to 20°C ~ 40°C in 20s by high-pressure helium, which further aggravated the damage of tumor cells. Multiple freezing and thawing of tumor by the argon-helium cryotherapy system can increase the freezing range, improve the ablation effect, and inactivate the tumor tissue in the target area.

Cryoablation may lead to tumor cell necrosis through a variety of mechanisms, including mechanical damage to the cell membrane after the formation of ice crystals by reducing temperature. Necrosis and apoptosis caused by cell injury stress, interruption of tumor blood supply during freezing, and the activation of anti-tumor antibodies to a certain extent (2). The microscopic changes in the cells treated with cryoablation begin with the formation of ice crystals in the cells, damage continuing with the thawing process. The formation of ice crystals leads to an increase in the volume of the cell contents, resulting in stress that damages the cell membrane, which in turn leads to cell necrosis and the release of intracellular contents. These releases can activate a certain amount of anti-tumor immune response, and thus the destruction effect of cryoablation on tumor cells to some extent. At the same time, the normal mitochondria function can also be disturbed during the freeze-thaw process. Mitochondrial dysfunction can cause apoptosis, so we can detect an increase in the pro-apoptotic Bax protein in these cells. During the process of cryoablation, not only the tumor blood vessels will be damaged, but also intravascular embolism will be caused, which will lead to a sharp reduction in the blood supply of the tumor and enhance the killing effect of cryoablation on tumor cells. Upon a decrease in temperature, the extracellular fluid of the tissue frozen, and the formation of extracellular ice increases the osmotic pressure outside the cell, resulting in a transfer of fluid from the inside to the outside of the cell (3).In this hypertonic environment, changes in intracellular pH and cellular contents can result in cell damage. The strength of these responses will affect the ablation effect. Some other important parameters will affect the ablation effect of cryoablation:1) The number and location of probes. 2) The shape of the ice ball covering the target tumor. 3) The depth of ice ball penetration. 4) The number of freeze thaw cycles. 5) The time of cryoablation (4).

## Clinical application of cryoablation

Compared with other ablation methods, cryoablation has obvious advantages, which can clearly show the shape of the frozen ice ball under ultrasound, CT, MRI and other imaging equipment. It is easier for the surgeon to control the ablation range. The patient has less pain and the operation is easier to be tolerated. Cryoablation has been used to treat tumors in many sites, including the lung, liver, kidney, breast, and prostate (**Table 1**).

**Table 1:** Cryoablation in different organs.

Target Organs	Number of articles	Benefits	Shortcomings
**Liver**	7	Relief of pain symptoms (5) Suitable for patients intolerant to surgery (6) Suitable for palliative care (6) Minimal effect on surrounding organs (7)	Less security than IRE (5) but better than RFA (8) Higher risk of complications in large tumors (9) Inflammation of appendages (10, 11)
**Lung**	8	Suitable for inoperable patients, lung function is well preserved (12) Suitable for palliative care (12) Minor complications are common and most require no intervention, major complications are rare (13)	Pneumothorax (13) Hemoptysis, hemothorax, pleural effusion and pulmonary infection (14) Cardiac arrest (15)
**Renal**	7	High overall survival (16–19) Low complications, well preserved renal function (20)	Pain and paresthesia (21) The efficacy is low on >2cm tumors (16, 22)
**Prostate**	6	Preservation of sexual function (23, 24) Short recovery time (25) Complications are minor and most require no intervention (26)	Urinary impairment and urinary tract infections (26) Recurrence after the first cryoablation (27) Complications of the second ablation increased (28)

IRE, irreversible electroporation; RFA, radiofrequency ablation.

### Liver cancer

Hepatocellular carcinoma (HCC) is the sixth most common malignant tumor in the world, and many patients are intolerant to surgical resection after diagnosis due to poor liver reserve function, multiple liver tumors, and extrahepatic diseases (29). In patients with HCC who are not suitable for surgery, the National Comprehensive Cancer Network(NCCN) recommends the use of local ablation, including cryoablation (6).

In patients with unresectable HCC, fewer complications occurred in the cryoablation group, although overall survival (OS) was not significantly different between the cryoablation and cryoablation combined with surgery groups (30). Local tumor progression (LTP) rates were compared between patients who underwent cryoablation and RFA for perivascular HCC at median follow-up. The cumulative LTP rates at 1 and 3 years were 8.3% and 17.3% in the cryoablation group, and 8.7% and 26.1% in the RFA group, respectively, with no significant difference (p = 0.379).Although the difference was not significant, vascular thrombosis and hepatic infarction around the cryoablation area were rare, and it can be considered that the efficacy and safety of cryoablation are better than RFA (8).Cryoablation (n=12) and irreversible electroporation (IRE) (n=12) were performed on 24 New Zealand rabbits with hepatic tumors located 0.5cm from the gallbladder. Gallbladder perforation was not observed in the IRE group, but only mucosal epithelial necrosis and serous layer edema were observed in the IRE group. Four rabbits suffered gallbladder perforation in the cryoablation group, and serum aminotransferase and bilirubin levels were higher in the cryoablation group than in the IRE group. Bile duct and granulation tissue proliferation was observed only in the ablation area of the IRE group. Therefore, the investigators concluded that IRE was safer (with or without gallbladder perforation) and more effective (faster recovery) than cryoablation (6). It can be seen from above that, although the safety and efficacy are not as good as IRE, they are better than RFA. The size of liver tumors affects the outcome of cryoablation. The effective rate of cryoablation is significantly reduced when liver tumors are larger than 4cm, accompanied by a higher probability of recurrence and complications (9).In the small area of cryoablation less than 3 cm, the incidence of complications was about 29%, and 87% of patients had no tumor progression at 6 months follow-up (7).In mouse models, cryoablation of over 30% of the volume of liver parenchyma volume resulted in an increased probability of systemic complications, including acute lung injury and increased pulmonary capillary permeability (10, 11).Patients with liver metastases have a high mortality rate and poor prognosis, and there is no standardized treatment for these patients. A retrospective study of 19 patients who underwent cryoablation for liver metastases after gastrectomy for gastric cancer found that the median OS was 16 months, and the median local tumor Progression-free survival(PFS) was 8 months. The quality of life of patients was improved after cryoablation (P <0.05). It can be concluded that cryoablation is an effective palliative treatment for liver metastases. It can improve the quality of life of patients and reduce the incidence of complications (5). Cryoablation not only has satisfactory therapeutic effects, but also has been shown to help patients relieve pain caused by liver metastases (31).This is of great significance for palliative treatment and improving quality of life in patients with metastatic tumors. Although the results of cryoablation for hepatocellular carcinoma are dependent on the size of the tumor, the overall results are satisfactory with fewer complications and are significant for the relief of symptoms in advanced patients.

### Lung cancer

Lung cancer is the most common cause of cancer-related death in adults (32). Lung cancer kills about 1.6 million people worldwide every year. In lung cancer, non-small cell lung cancer (NSCLC) accounts for 75%-80%. Among them, stage I and II accounted for a small number of patients, who had the opportunity to be operated, and the 5-year survival rate was high. Most of the patients were in stage III-IV, and the 5-year survival rate was only 12% with palliative treatment alone. Although stage IIIA patients can still undergo surgery, the recurrence rate is high, and the 5-year survival rate is only 10% to 30% (33).For these patients, traditional treatment would consider chemotherapy and radiotherapy, which not only have no significant efficacy, but also are accompanied by serious adverse reactions. In 1998, researchers began to study the application of cryoablation in the minimally invasive treatment of NSCLC tumors. Because of its safety, high efficiency, minimal invasion and few side effects, percutaneous cryoablation has been an important treatment option for unresectable primary and secondary peripheral lung tumors for more than ten years (34).

Surgery is often the choice for early-stage lung cancer, but cryoablation is now a reasonable option. Percutaneous cryoablation was performed in 22 patients with stage I NSCLC (34 tumors), and only one tumor progression was detected after a median follow-up of 23 months. There were three deaths, one of which was due to lung cancer. The 2 and 3-year overall survival (OS) rates were both 88%, and the disease-free survival (DFS) rates were 78% and 67%, respectively (35). Cryoablation for advanced lung cancer can achieve tumor reduction, relieve symptoms and improve the quality of life. A total of 54 patients with stage IV lung cancer were followed up for 6.5 years, and the OS of cryoablation group was significantly longer than that of palliative group (median OS:14 months vs 7 months, P = 0.0009).It is reasonable to assume that there are significant differences between cryoablation and palliative care (36). At the same time, it was found that multiple ablation groups (2,3,4 freeze-thaw cycles) had a significant effect on improving OS. The incidence of recurrent or primary lung cancer in the residual lung after pneumonectomy has been reported to be high (21%-44%) (37). Because of the significant risk of loss of lung function after pneumonectomy and their poor physical condition, such patients often cannot tolerate lung cancer resection. However, percutaneous cryoablation can be used to remove lung tumors while preserving most lung function (12).Percutaneous cryoablation can also be considered for the treatment of lung tumors in patients who have undergone pulmonary resection.

### Renal cancer

Open partial nephrectomy (PN) is recognized as the standard treatment for renal tumors (38).Laparoscopic PN is widely used, and the incidence of complications is higher than that of open PN (39).Cryoablation has the advantages of laparoscopic minimally invasive surgery, but its complications are lower than those of laparoscopic PN (40). It is suitable for patients with poor general condition or multiple renal tumors, and the efficacy is equivalent to that of nephrectomy (16). In a rabbit model of transplanted renal tumors, there was no significant difference in disease-free survival at 28 days between cryoablation and radical nephrectomy, suggesting that both treatments are equally effective *in vivo (* 41). Similarly, a study comparing percutaneous cryoablation with partial nephrectomy in 118 patients with primary solitary renal tumors found no significant differences in complication rates, preservation of renal function, recurrence rates, or cancer-specific mortality between the two procedures (42). Similar rates of OS and complications were found in cryoablation of small tumors less than 4 cm in diameter (17, 18, 43). At the same time, the damage of surrounding organs caused by surgery was evaluated. It can be argued that cryoablation of renal tumors results in a higher overall survival rate, a lower incidence of postoperative complications, and better preservation of renal function compared with partial nephrectomy (20).The efficacy of cryoablation of renal tumors is related to the size of the tumor. Studies have compared outcomes after cryoablation in patients with tumors less than 3 cm (mean diameter 1.8 cm) and at least 3 cm (mean diameter 4 cm). Histopathological examination showed 46.7% Renal cell carcinoma(RCC) in the < 3 cm group versus 66.7% in the ≥ 3 cm group. Only patients with tumors in the at least 3 cm group experienced complications (62%) and death (two patients) (19). The outcomes of renal-tumor ablation are related to stage. Cryoablation of T1 renal masses was associated with lower overall and postoperative complication rates and better preservation of renal function than PN. For T1b renal tumors, cryoablation was associated with a 2.5-fold increase in 5-year cancer-related mortality compared with PN (22). Although cryoablation of renal cancer is related to tumor size and stage, cryotherapy is an important treatment for renal cancer with the same efficacy and fewer complications as open PN.

### Prostate cancer

Prostate cancer (PCa) is the second most common cancer in men, generally 90% of it confined to the prostate gland. Conventional treatment of PCa often leads to functional complications. Due to the advances in imaging, cryoablation therapy has developed rapidly, providing a new option for PCa patients. Compared with radical surgery, cryoablation has the characteristics of faster recovery of daily activities, shorter hospital stay, lower postoperative recurrence and lower overall treatment cost (25). Focal cryoablation in the treatment of mid-stage prostate cancer can show obvious advantages in preserving urinary and sexual function (23). Studies have shown that although urinary retention and urinary tract infection are common complications of PCa cryoablation, cryoablation safety is clinically acceptableand 91% of minor complications do not require any intervention (26). In a retrospective study of 82 cases, partial prostate cryoablation was associated with excellent oncologic and functional outcomes in men with localized PCa (24). It has been shown that after the first round of radiotherapy (28) or after primary cryoablation (27), cryoablation is effective for recurrent prostate tumors. There was a slight increase in complication rates after the second cryoablation compared with the first cryoablation for prostate cancer.

## Cryoablation results in changes in immune function

Cryoablation can cause necrosis of tumor cells, but also can cause immune targeting stimulation of tumor cells. These immune responses occurred after ablation-induced tumor cell death, but not in the case of surgical removal of the tumor (44).Compared with conventional cancer therapy, cryoablation has fewer adverse reactions and can promote a more comprehensive and effective release of autologous antigens into the circulation. Early studies reported immune-mediated responses to metastases from the primary tumor after ablation (45). In the mouse MT901 breast cancer cells cryoablation using the tumor rechallenge model, it was found that the tumor recurrence rate of mice after surgical resection alone was 86%, while the tumor recurrence rate of mice after cryoablation was only 16% (46). The authors speculated that it might be due to the large amount of tumor-specific autoantigens released from necrotic tumor cells caused by cryoablation. Similarly, serum Prostate specific antigen (PSA) has been reported to increase in prostate cancer after cryoablation (47). The increased release of these neoantigens would lead to increased danger signals, which would initiate the process of enhancing cancer immunity. There is no consensus on the secondary cause of cell death for immune changes after cryoablation. Some think it is secondary to mechanical force induced cell necrosis, some to MLKL and RIP kinase phosphorylation necrosis, and some to thrombosis and other vasogenic factors or a combination of these above factors. The more widely accepted theory of the underlying mechanistic association between cryoablation and the immune system is Matzinger’s dangerous theory (48). She proposed that cells secrete danger signals after they die, and these signals can lead to a range of immune responses. Cell death caused by cryoablation with cell contents remaining intact, which induces the release of intracellular DNA, RNA and heat shock proteins (HSP) (49). Similarly, the heat shock protein HSP70 has been reported to increase after cryoablation of melanoma (50). HSP70 completes antigen presentation by chaperoning antigen to dendritic cells(DC) and promoting the expression of major histocompatibility complex class 1 (MHC 1) (51). This process can allow DC to mature and thus fully activate T cells, leading to specific tumor immune responses. The above changes in DC during antigen presentation have been observed in various cancers during cryoablation. Interestingly, in the melanoma mouse model, antigen accumulation in DC after cryoablation was significantly greater than that after RFA (52).

The targeted migration of activated tumor-specific T cells is associated with Chemokine ligand 21 (CCL21) and Intercellular adhesion molecules 1 (ICAM-1). CCL21 and ICAM-1 were overexpressed in CD31-positive endothelial cells in tumor tissues and endothelial venules of tumor-draining lymph nodes, and an increase in naive CD8+T cells can be found in tumor tissues and draining lymph nodes. It was noted in the mouse studies that T-cell migration began to increase as early as 1 hour after ablation and reached a maximum after 6 h (53). It results in an increase in tumor-specific lymphocytes.

Many studies have pointed out that the specific lymphocytes activated after ablation are mainly CD4+ and CD8+T lymphocytes (54–56). Ablation studies in colorectal cancer have found that Programmed cell death 1 ligand 1 (PD-L1) expression in target tumor tissues and programmed death 1 (PD-1) expression in specific CD8+ and CD4+T lymphocytes are increased (57). At the same time, natural killer (NK) cells and macrophages increased (58). Studies have reported similar results, in addition to tumor regression, decreased lung metastasis, and increased systemic CD4+ and CD8+T cells, cryoablation was also accompanied by an increase in NK cells (59). With the increase of CD4+ and CD8+ specific T cells, studies on HCC and other cancers have found that ablation will lead to the reduction of immunosuppressive regulatory T cells (Treg) (60, 61). The increase of CD4+ and CD8+ T cells and the decrease of Treg cells after cryoablation are important for tumor control and patient survival (60). In studies comparing cryoablation and RFA, although both can induce antigen-specific T lymphocyte responses, cryoablation can induce stronger antigen-specific CD4+T cells, so cryoablation has a stronger ability to induce anti-tumor immune targeting (62).

Cryoablation also affects cytokine levels, which also affects the immune response. At the same time, tumor tissue, age, and freezing speed all affect the immune process (63).The increase in interferon-γ (IFN-γ) levels after ablation suggests that the upregulation of T helper 1 (Th1) cell responses is more related to antibody-dependent cell-mediated cytotoxicity (ADDC) responses (64). In addition, the proinflammatory cytokines interleukin-1β (IL-1β), interleukin-6 (IL-6) and interleukin-8 (IL-8) were all increased to varying degrees after thermal ablation (65, 66). Increases in these cytokines contribute to specific T-cell activation and Th1 responses. Interestingly, it has been reported that during liver cryoablation, when more than 20% of the liver is ablated, the release of cytokines IL-6, IL-10 and Tumor necrosis factor α (TNFα) may induce systemic inflammatory response, which may lead to systemic effects (67). At this point, cryoablation should be replaced by other thermal ablation that is more effective (68). Studies have shown that these markers of inflammation and cell damage are significantly elevated after liver cryoablation in normal mice and are higher than other ablation methods (67).

Cryoablation has been shown to enhance the immune response in many studies, but it does not last long, about 4 weeks. Studies have shown that specific T cells have anti-tumor cytolytic effects 2 to 4 weeks after ablation (69). Specific T cells persisted during these 4 weeks (70).

In A clinical study of 22 patients with renal cell carcinoma before and 3 months after cryoablation and blood samples of some patients, it was found that CD8+T, CD4+T, granzyme A(GZMA), CD11c transcription levels were significantly increased, and CD8/FOXP3 was increased after cryoablation. In addition, T-cell receptor β (TCR-β) full spectrum analysis showed that cryoablation caused expansion of some T cells in tumor tissue, which also supported this immune stimulation process (71).

Many animal experiments have found that cryoablation not only induces immune stimulation, but also induces immunosuppression (49). In the rat fibrosarcoma model, the early stage of cryoablation increases the probability of lung metastasis, and the anti-tumor activity of spleen after cryoablation is lower than that of surgical resection at different time points (72). When the area of cryoablation was large or multiple nodules were cryoablation, the anti-tumor immune response was weaker than that of a single liver nodule (73, 74). Cryoablation was performed in a rat tumor model, it was found that if most of the necrotic tumor tissue after freezing remained in the animal, the immune response can be suppressed. On the contrary, leaving only a small amount of frozen tissue, the tumor growth is inhibited or even remission, suggesting that there is a threshold of antigen stimulation, and excess antigen may induce immunosuppressive response. From the above reports, we can speculate that there is immunosuppression after cryoablation in some cases. At present, the mechanism of immunosuppression is not clear, but some studies have shown that it may be related to the ratio of necrosis and apoptosis and the excessive production of Treg (49, 75, 76). No danger signals will be released when the tumor tissue dies due to apoptosis, so the DC without antigen presentation will not mature. Here, if macrophages are primarily responsible for TNF uptake, this may bias the immune response towards humoral rather than cellular immunity because macrophages do not cross-present antigens as dendritic cells do. Macrophages may release IL-10 or TGF-b, further weakening the T cell response. Immature DC cannot promote the release of cytokines by TH1 cells, TH2 cells and other cells, so it cannot continue to activate T cells (CD8+T, CD4+T cells) to produce immune response. Tregs, by contrast, are at an advantage. At the same time, it also induces an increase in Treg number, an increase in inhibitory cytokines and a decrease in stimulatory cytokines, leading to an inhibitory immune response (49).On the contrary, necrosis causes the release of danger signals, DC can mature, then stimulate the production of tumor specific T lymphocytes and produce immune stimulation. Therefore, when apoptosis is dominant in the ablated tissue, the overall performance is immunosuppression.

## Tumor immunotherapy

Tumor immunotherapy, which can be divided into active immunity and passive immunity, aims to control and eliminate tumor cells by enhancing autologous anti-tumor immune response. Active immunization mainly acts on the immune system. For example, cytokines that enhance the body’s immune function, antigen-dependent therapeutic vaccines, and non-antigen-dependent inhibitors that regulate T cell function all work through active immunity. Passive immunity acts directly on tumor cells. Such as anti-tumor monoclonal antibodies and adoptive immunotherapy work through passive immunity. At present, the main clinical immunotherapy strategies include immune checkpoint blockade, TLR agonist therapy, adoptive cellular immunotherapy, tumor vaccines, oncolytic viruses, antibodies or recombinant proteins, etc.

Immune checkpoint blockade is considered to be the most promising treatment. Immune checkpoint refers to molecules on the surface of immune cells that transmit inhibitory expression signals. Under normal circumstances, it can protect the body from severe autoimmune response, but it can enable tumor cells to successfully escape from the immune system attack in the tumor microenvironment. At present, the most studied immune checkpoint molecules are Cytotoxic T-lymphocyte-associated antigen 4 (CTLA-4) and PD-1. By blocking the corresponding immune checkpoint signaling pathways, the activation and proliferation of T cells can be restored and the anti-tumor immune response can be enhanced (77). PD-1/PD-L1 and CTLA-4 antibodies, which are representative of immune checkpoint inhibitors (ICIs), are considered to be revolutionary advances in oncology and have been approved by the US Food and Drug Administration (FDA) for the treatment of malignant melanoma, lung cancer, liver cancer, gastric cancer, urothelial cancer and lymphoma (78). Blockade of the CTLA-4 interaction site by antibodies to CTLA-4 allows CD80/86 ligands to be used with CD28 to activate T cells (79, 80).CTLA-4 antibody can also inhibit the function of Treg expressed on Th cells (81). PD-1 is a key immune checkpoint molecule expressed primarily on T lymphocytes. It inhibits the cytotoxic capacity of T lymphocytes by binding to its ligand PD-L1, which has been observed in increased numbers in several malignancies and can promote tumor immune escape and T lymphocyte exhaustion (82). Antibodies that block PD-1 or PD-L1 are designed to restore depleted T cells. The emergence of immunotherapy has dramatically changed the treatment landscape of advanced NSCLC. ICIs targeting the PD-1/PD-L1 pathway are now first-line treatments for stage IV NSCLC (when tumor PD-L1 expression is ≥ 50% and no molecular oncogenic target is present) (83). However, the response rate is only about 20% in most clinical trials (84). The reason may be related to less Tumor Infiltrating Lymphocytes (TIL) in the tumor microenvironment (39). The combination of CTLA-4 and PD-1 blockade has been shown to improve survival in patients with melanoma, renal cell carcinoma, and many other cancers (85, 86).

Toll-like receptors(TLR) agonist Therapy. TLR is a type of pattern recognition receptor expressed on macrophages and DCS. TLR signaling triggers a metabolic shift in macrophages and DCS that promotes the transition from a tolerogenic to an immunogenic state. When TLR7 and TLR9 were combined with pathogen-associated molecular pattern (PAMP) unmethylated CpG or the agonist imiquimod respectively, they stimulated DC and activated TH 1 cells. Thus, it promotes Cytotoxic Tlymphocyte activity (81).

Adoptive T-cell transfer (ACT) therapy involves first isolating lymphocytes from a patient’s tumor tissue, draining lymph nodes, or peripheral blood, expanding them *in vitro*, then infusing the patient to destroy systemic tumor cells (87). Chimeric antigen receptor (CAR) T cells have emerged, and autologous T cells designed to express a CAR specific for the CD19B lymphocyte molecule have recently been approved by the FDA for the treatment of refractory pre-B-cell acute lymphoblastic leukemia and diffuse large B-cell lymphoma (88). Recent studies have shown that TIL can target neoantigens in melanoma, which confirms the effect of TIL therapy (89). CAR-NK cells are more efficient because of their ability to recognize tumors through both CAR and natural NK cell receptors. In addition, NK cells can be transplanted from an allogeneic source and so are more readily available, making it less susceptible to eliciting a graft-versus-host immune response (90).

Epigenetic regulation of DNA methylation, non-coding RNAs, and histone modifications plays a key role in the mechanism of cell growth and differentiation (91). Small-molecule inhibitors of DNA Methyl transferase(DNMT) exert anti-tumor effects, such as apoptosis, cell cycle arrest and differentiation, by re-expressing genes that have been silenced by DNA methylation.

Tumor vaccines can specifically activate host T cells to combat tumor antigens, but their efficacy is not ideal due to the difficulty of antigen selection and the lack of understanding of tumor immune mechanism. Oncolytic virus immunotherapy uses natural or synthetic viruses to selectively replicate and kill cancer cells. Oncolytic virus T-VEC is currently used in clinical practice for advanced melanoma. It is a modified herpes simplex virus type I, which has the advantage of killing tumor cells without damaging neurons (92).

### Synergistic antitumor effects of immunotherapy and cryoablation

As mentioned in the beginning of the article, cryoablation is considered to be the main consideration for combination immunotherapy because imaging monitoring can better control the ablation area and guide the ablation needle, reduce complications, surgical costs, and patient recovery time (93).Although all ablation techniques result in release of tumor antigens in situ, cryoablation is superior to other ablations in preserving the native antigen structure (94). Tumor thermal ablation produces degenerative antigens, but adaptive immunity from tumor non-degenerative antigens is more likely to produce off-range effects. Therefore, cryoablation is more suitable for use in combination with various immunotherapies. Moreover, the immune response stimulated by ablation alone is usually too weak to completely eliminate the target tumor, so it is also necessary to combine cryoablation with immunotherapy (94, 95). To enhance the immunogenic effect of cryoablation, different immunotherapies can be administered, either to enhance innate immunity or to enhance specific T-lymphocyte activity. When the contents of tumor cells are released after cryoablation, different stages of content presentation are enhanced by different immune agents. The result is a stronger specific tumor immune response.

This antitumor effect was reported to be further enhanced when cryoablation was used in combination with CTLA-4 antibody, with up to 80% of mice becoming tumor-free (52). In a mouse model of prostate cancer, cryoablation combined with anti-CTLA-4 antibody treatment prolonged the survival time of the mice by 14.8 days. Meanwhile, the mortality rate was 4-fold lower than that of cryoablation alone, suggesting that anti-CTLA-4 antibody can significantly enhance the immune response mediated by cryoablation (96). Also in the mouse model of prostate cancer, the combination of CTLA-4 antibody and cryoablation could effectively inhibit or slow down the growth of secondary tumors or induce tumor rejection (97). In the treatment of mouse renal cell carcinoma by cryoablation combined with PD-1 inhibitor, the inhibitory effect of combined therapy on tumor metastatic growth was significantly stronger than that of drug administration alone or freezing alone. Meanwhile, PD-1 inhibitor significantly enhanced the immune response induced by freezing (including increased number of CD8+TIL, mRNA expression levels of INF-γ and GZMB) (98). Therefore, we can assume that cryoablation combined with CTLA-4 or PD-1 inhibitors can enhance the anti-tumor effect and may also reverse the tumor effect. The 5-year recurrence-free survival rate was 91% in patients treated with cryoablation plus imiquimod(TLR7/8 agonist) (99). In a mouse model of melanoma, antitumor immunity after cryoablation can be enhanced by coinjection of saponin-based adjuvant and TLR9’s adjuvant CpG to affect IL-1 production and the number of multifunctional T cells in draining lymph nodes (100). The efficacy of TLR agonists combined with cryotherapy was also satisfactory. Cryoablation joint sorafenib treatment of advanced renal cell carcinoma of the efficacy and safety, found that patients with combination therapy on drug response rate, disease control rates were significantly higher than accept sorafenib monotherapy. It’s PFS and OS were significantly longer than the sorafenib monotherapy, immune related indexes were significantly improved without increaseing adverse reactions (101). The effect of cryoablation combined with IL-12 on anti-tumor immunity of rat glioblastoma xenograft was observed. It was found that the cellular immune function of cryoablation combined with IL-12 was significantly enhanced, the tumor regression was observed, and the subsequent immune activity was significantly enhanced. The number of CD11c+ DCS and CD86+ lymphocytes was significantly increased, and the expression of IFN-γ was also significantly increased (102). A pioneering study of mammary gland carcinoma in mice showed that the tumor drainage lymph nodes for the adoptive transfer of tumor-specific T cells, increase in the number after cryoablation. Compared with adoptive transfer of T cells that were surgically removed or controlled and then collected from tumor-draining lymph nodes, this resulted in significantly fewer lung metastases (103). Two clinical studies reported favorable overall survival results when cryoablation was combined with immunotherapy with allogeneic NK cell infusion and CD factor-induced killer (DC-CIK) cells (104, 105). After cryoablation and allogeneic NK cell transfer, survival and immune response outcomes in patients with lung, renal, and hepatocellular cancers have been relatively satisfactory (105–107).. In addition, patients who received multiple NK-cell transfusions showed better PFS than those who received a single transfusion (105). Due to the relative ease of operation of ACT therapy, a growing number of combined cryotherapy studies have also reported satisfactory results.

## Conclusion

The literature review strongly supports the immunorecognition of tumor-specific antigens and the effect to distant lesions. The evidence covers clinical cryoablation, various animal models and studies of immunity in patients undergoing cryoablation. Although immunosuppressive responses may occur with combination therapy, the immune system generally presents a satisfactory antitumor outcome. Above studies preliminary clinical data proves the cryoablation, with the combination of immune therapy can enhance innate immunity and tumor specific T lymphocyte activity, to help the immune system against cancer. So there is reason to believe that cryoablation, has the very good synergy between and immune therapy.

Several important questions remain to be addressed:

- What ratio of necrosis to apoptosis is best for our treatment of tumors? How do we get to that ratio?
- What are the characteristics of the tissue and environment in which immunosuppressive reactions occur? Is there a way that we can avoid it and enhance the anti-tumor response?
- Due to the immune system itself and individuals vary so much, immunotherapy can vary from tumor to tumor or from individual to individual. This is the biggest challenge facing cryoablation immunity in the future.

We can better optimize treatment when we understand the immune response caused by cryoablation. It helps further optimize clinical application to achieve the goal of minimal side effects and maximum efficacy. Before clinical promotion of cryoimmunotherapy for different tumors, sufficient animal experiments and data support must be done to determine the application conditions and ensure the safety of clinical application. In the future, cryoimmunotherapy should be personalized according to the immune characteristics of tumors and patients, prepare preparatory plans, and cope with reaction complications, so as to maximize its effect.

## Author contributions

ZC conducted the analyses and drafted the manuscript. JZ contributed to and analysis. LM contributed to study conception and design. All authors contributed to the article and approved the submitted version.
